# Macrocell Corrosion of Steel in Concrete under Carbonation, Internal Chloride Admixing and Accelerated Chloride Penetration Conditions

**DOI:** 10.3390/ma14247691

**Published:** 2021-12-13

**Authors:** Zhilu Jiang, Siyao Li, Chuanqing Fu, Zheng Dong, Xuefeng Zhang, Nanguo Jin, Tian Xia

**Affiliations:** 1 College of Civil Engineering, Zhejiang University of Technology, Hangzhou 310014, China; zljiang@zjut.edu.cn (Z.J.); lisiyao@zjut.edu.cn (S.L.); zdong@zjut.edu.cn (Z.D.); 2 Key Laboratory of Civil Engineering Structure & Disaster Prevention and Mitigation Technology of Zhejiang Province, Hangzhou 310014, China; 3 Engineering Technology R&D Center, Zhejiang University of Technology Engineering Design Group Co., Ltd., Hangzhou 310014, China; chafu@zjut.edu.cn; 4 College of Civil Engineering and Architecture, Zhejiang University, Hangzhou 310027, China; jinng@zju.edu.cn (N.J.); zljiang@szu.edu.cn (T.X.)

**Keywords:** corrosion of steel, concrete, chloride penetration, carbonation, macrocell corrosion

## Abstract

Steel corrosion has become the main reason for the deterioration of reinforced concrete structures. Due to the heterogeneity of concrete and the spatial variation of environmental conditions, macrocell corrosion is often formed by localized corrosion, which is more detrimental if the anode is supported by large numbers of cathodes. The macrocell corrosion caused by concrete carbonation has been seldom studied. Furthermore, the influence of geometrical conditions on cathode-controlled corrosion in the chloride environment needs to be further clarified. In this work, the macrocell corrosion of steel embedded in concrete specimens exposed to accelerated carbonation, chloride contamination, and chloride penetration is studied using a modified ASTM G109 method. Concrete specimens with various binder types, geometrical parameters (i.e., concrete cover thickness and the diameter of embedded steel), and boundary conditions were tested. A simplified mathematical model for the prediction of the steel corrosion rate was developed considering two-dimensional oxygen diffusion. The results showed that, at the same level of anodic potential drops, the corrosion current rate in chloride-induced corrosion is higher than that of carbonation-induced corrosion. Chloride contamination is less detrimental to concrete incorporated with slag and pulverized fly ash than it is to pure ordinary Portland cement (OPC), likely due to enhanced chloride binding capacity. The results also indicated that the model considering two-dimensional diffusion can accurately predict the cathodic reaction process on corroded steel bars, which provides a theoretical basis for considering the correction coefficient of steel bar position in the establishment of a steel bar corrosion rate model.

## 1. Introduction

The chloride- and carbonation-induced corrosion of steel reinforcing bars is the main reason for the deterioration and premature failure of concrete structures [1]. The corrosion initiation of steel (i.e., depassivation) begins either when the chloride concentration in the pore solution (i.e., free chloride) on a steel surface reaches a threshold value or as the pH drops below a critical value due to carbonation [2,3,4,5,6]. The chloride-induced corrosion is particularly concerned with structures exposed to marine or deicing salt environments, while carbonation-induced corrosion affects a wider range of structures. Expansive rusts formed due to corrosion propagation can generate longitudinal cracks in concrete cover, degrade the bonding between reinforcement and concrete, and reduce the bearing capacity of structural members [7,8,9,10]. Therefore, illuminating the steel depassivation and corrosion propagation process in concrete exposed to different environmental conditions can provide important information on selecting appropriate corrosion control and monitoring measures.

The development of corrosion rate under the carbonation effects is still under study. Generally, two main significant factors for carbonated concrete, i.e., the pore structure and water saturation, are related to the corrosion rate [11]. Some results [12,13] showed that the corrosion rate was reduced at a relative humidity (RH) above 90% due to limited oxygen diffusion at cathode. On the other hand, other research results [14] indicated that the corrosion rate continuously increased at an RH up to 99%. It can be explained by the incomplete filling of water in pores even at a high RH. Some large pores in concrete can only be saturated through capillary water absorption or immersion under water. This may imply that in a cyclic wetting–drying condition, oxygen-concentration controls cathodic reaction, which occurs as pores are saturated during the wetting period, and the corrosion rate decreases during drying period [15]. Carbonation-induced corrosion is commonly considered to be relatively homogeneous [16]. However, due to the concrete heterogeneity, such as complex pore structure, random aggregate distribution, and cracks, the carbonation depth is not uniform [17,18], and different corrosion conditions are produced along the steel bar, resulting in ‘macrocell corrosion’ [19]. The evolution of the corrosion rate of macrocell corrosion under a carbonation condition needs to be further studied.

There is still no consensus on the mechanism of steel depassivation due to chloride ions. One model [20] regarded depassivation as the adsorption and penetration of chloride ions through the film layer, which was confirmed by the modification of the passive film exposed to simulated pore solutions by a recent experimental study [21,22]. The other model [23] suggested that the chloride ions stayed on the film surface and acted as a catalyst in depassivation. This theory was supported by a molecular dynamics simulation [24] in which the penetration of chloride in the film was not found. In practice, the critical chloride content is often used to identify steel depassivation. However, the critical chloride content varies by a wide range, depending on many material and environmental parameters [25,26,27]. Moreover, the difference between critical values from the laboratory and field conditions should be noticed [5]. Chloride-induced corrosion often forms small pits due to the localized distribution of chloride ions. Pit growth is an autocatalytic process if a sufficient [Cl^−^]/[OH^−^] ratio is achieved. Microcells are often observed in chloride-induced corrosion, leading to the local steel thinning and pit extension [28]. In particular, the distance between anodes and cathodes in a macrocell can be a few meters across the structure members [29]. When the reinforcement at one side of a concrete structure is depassivated (i.e., anode) while the opposite reinforcement with sufficient oxygen supply acts as cathode, corrosion macrocell is sustained. The current density in a macrocell is dependent on the geometry of the structure setup [29]. For a simplified mathematical function for the cathode-controlled current density [30], one-dimensional oxygen diffusion based on Fick’s law is often used which assumes gas diffusion is an ideal diffusion perpendicular to the surface of the plate electrode. However, the steel bars in concrete members are cylindrical, instead of flat. In addition, the influence of steel bars at different positions in concrete on the corrosion rate is not considered.

In concrete structures, macrocell corrosion consists of spatially isolated anodes and cathodes, while microcell corrosion contains pairs of closely adjacent anodes and cathodes of the same metal. Nevertheless, macrocells can also form on an individual bar with parts being exposed to various environments or with different compositions [31,32]. In comparison to microcell corrosion which often causes uniform corrosion, macrocell corrosion leads to pitting (localized) corrosion, which is more detrimental if the anode is supported by large numbers of cathodes. In previous laboratory studies [33,34], the macrocell measurements were typically commenced on ASTM G109 specimens, which were limited to the case of external chloride penetration. Nevertheless, the ASTM G109 test could be modified to study macrocell corrosion in concrete exposed to other environments or with various geometrical parameters and boundary conditions.

This study aims to study the macrocell corrosion behaviors of steel embedded in concrete exposed to three simulated environments, i.e., accelerated carbonation, chloride salts contamination, and cyclic drying–wetting chloride penetration. In addition, the influence of concrete cover thickness, steel diameters, and the exposed surface of specimens on the macrocell corrosion behaviors of steel is discussed. Finally, a mathematical model for the prediction of steel corrosion rate was developed considering two-dimensional oxygen diffusion and it was validated by the experimental results.

## 2. Experimental Programs

### 2.1. Materials and Mix Proportion

Concrete specimens with the same water-to-binder (w/b) ratio but different binder types were studied, including pure ordinary Portland cement (OPC), OPC blended with ground granulated blast-furnace slag (GGBS), OPC blended with Class F (low calcium), and pulverized fly ash (FA). The GGBS and FA substituted the OPC by 50% and 30% (by mass) in concrete, respectively, as listed in Table 1. The OPC with a 28-day compressive strength of the prepared hardened pastes of no less than 52.5 MPa was used. The OPC was composed of 55.5% (by mass) C3S, 19.1% C2S, 6.5% C3A, 10.1% C4AF, and 5% gypsum with the equivalent alkali content (denoted as Na_2_O + 0.658K_2_O) less than 0.60%. The fineness of OPC, GGBS, and FA was 350 m^2^/kg, 450 m^2^/kg, and 400 m^2^/kg, respectively. The sand was river sand with a fineness modulus of 2.64 and a moisture absorption capacity of 1.2%. The coarse aggregate was crushed gravel with a particle size of 5–20 mm. The water was from tap water.

### 2.2. Strength and Porosity

The compressive strength of concrete was measured on cubic specimens with a side length of 100 mm at 7, 14, and 28 days after casting. The porosity of concrete at the age of 7, 14, and 28 days was calculated following:

(1) $$Porosity(\%)=\frac{\left(m_{0}-m_{i}\right)}{V_{0}\cdot \rho_{w}}\times 100\%$$

in which *m*_0_ is the saturated mass of the concrete at the desired curing age; *m_i_* is the mass of oven-dried concrete at 105 ± 5 °C till constant readings were achieved; *ρ_w_* is the density of water; *V*_0_ is the volume of the concrete specimens.

### 2.3. Carbonation Resistance

The carbonation depth was measured on concrete specimens with a dimension of 100 mm × 100 mm × 400 mm after being exposed to a chamber environment at 20 °C, 70% RH, and 20% CO_2_ concentration for various durations. The carbonation depth was determined via spraying phenolphthalein on a freshly broken surface at the section of 100 mm × 100 mm, followed by callipering the width of the uncolored region vertically to the exposed surface. Due to the presence of aggregates and the random nature of the carbonation process, the carbonation depths from the surfaces were not uniform. The carbonation depth was calculated as the average of about 40 different measuring points along the edge of the broken surface. After each measurement, the broken surfaces were sealed with wax and exposed back to the carbonation chamber until the next carbonation time. For the concrete specimens moist cured for 28 days and carbonated for 126 days, the concrete powders were collected at the exposure surface. The powders, passing through a 0.63 mm sieve, were oven-dried at 105 ± 5 °C for 2 h (DHG-9000, YIHENG, Shanghai, China), followed by cooling down to room temperature. The powders were diluted with deionized water 10 times for 24 h, and the pH in the solution was measured using a PHS-3C pH meter.

### 2.4. Chloride Penetration Resistance

**Figure 1:**
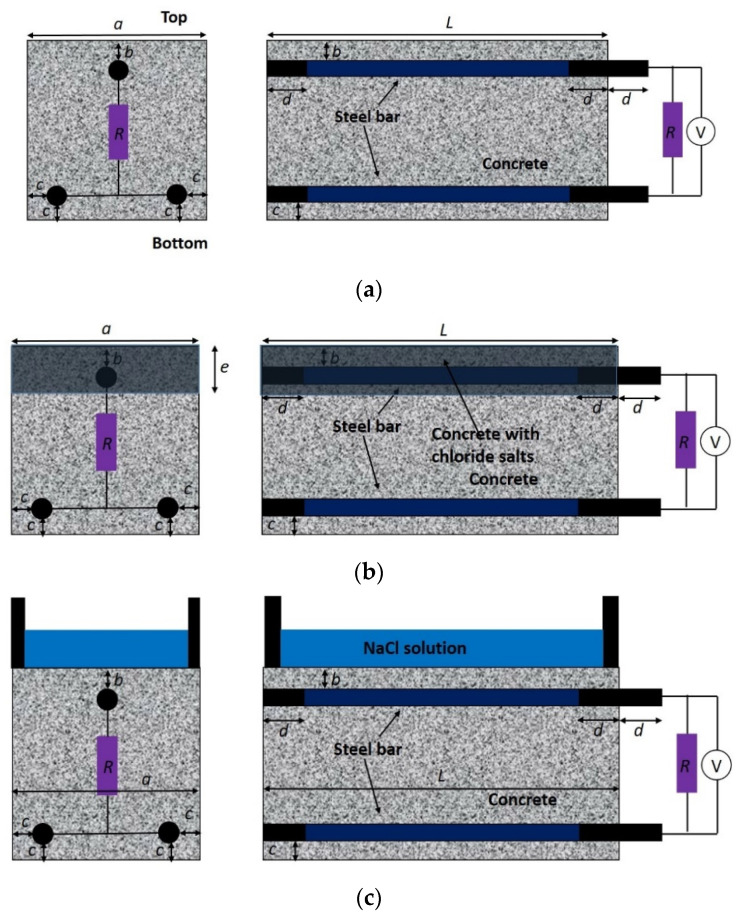
Configuration of modified ASTM G109 specimens to simulate the macrocell corrosion in concrete structure exposed to (**a**) accelerated carbonation; (**b**) internally admixed chloride; (**c**) cyclic drying–wetting chloride penetration. (a = 150 mm, b = 10 mm, c = 15 mm, L = 250 mm, d = 25 mm, and e = 30 mm).

The corrosion resistance of concrete was assessed by measuring the time-dependent anodic corrosion potential and macrocell corrosion current of steel embedded in concrete specimens using a modified ASTM G109 method [35]. The configurations of the specimens are illustrated in Figure 1. In particular, three reinforcing steel bars with a diameter of 16 mm were embedded in each concrete specimen with a dimension of 250 mm × 150 mm × 150 mm. Two bars were positioned at the bottom serving as the cathode, while one bar was positioned at the top serving as the anode. The two ends of the bars were epoxy coated to prevent extraneous effects, leaving 200 mm of contact length in the middle with concrete. The concrete cover depth was 10 mm for the top bar and 15 mm for the bottom bars. After 28 days of moist curing, four lateral surfaces of specimens were sealed with wax, leaving the top and bottom surfaces (250 mm × 150 mm) exposed. The two bottom bars were connected and further connected to the top bar through a 100 Ω resistor using wires. The macrocell corrosion current of the specimens was calculated using Ohm’s law by monitoring the voltages at the two ends of the resistor.

The concrete specimens were exposed to three different simulated environments: (i) accelerated carbonation, (ii) internally admixed chloride, and (iii) chloride penetration under drying–wetting cycles. For the specimens corroded under accelerated carbonation, they were stored in a carbonation chamber at a temperature of 20 °C, humidity of 70%, and CO_2_ concentration of 20%, with the bottom surface of the specimen covered with parafilm during carbonation. This treatment was to ensure that the bottom bar remained passive while the top bar was gradually depassivated by carbonation. For the specimens exposed to chloride contamination, the top layer of concrete with a thickness of about 30 mm was internally admixed with 0%, 1.5%, 3%, or 4.5% NaCl with respect to the cement mass. This treatment also allowed for the immediate activation of the top bar while keeping the bottom bars passive. For the specimens subjected to chloride penetration, a pond with a dimension of 200 mm × 75 mm × 40 mm was mounted to the top surface of the specimens. The chloride penetration from the pond into the top layer of the specimen was accelerated by the drying–wetting cycle, which was composed of an immersion in 3% NaCl solution for 2 weeks and atmospheric drying for 2 weeks. During the wetting stage, the pond was sealed to minimize evaporation. The specimens in Figure 1b,c were padded at the bottom to ensure the accessibility of oxygen into the bottom surface of the specimens. During the exposure of specimens in these three simulated environments, the anodic potential of the top bar was periodically monitored. In particular, the top steel bar served as the working electrode, an SCE (saturated calomel electrode) served as the reference electrode, and stainless steel plates were an auxiliary electrode.

To study the effect of the geometrical parameters and boundary conditions of the specimen on the macrocell corrosion behavior of steel in concrete, the same configuration (see Figure 1b) but with different concrete cover thickness, steel diameters, RH, and sealed conditions was adopted. In particular, concrete specimens with three different values of cover thickness at the bottom (10 mm, 15 mm, and 20 mm) and three steel diameters (10 mm, 15 mm, and 22 mm) were prepared. The depassivation of the top layer of steel was induced by admixing the top layer of concrete with 3% NaCl. The concrete specimens were placed in a chamber at 20 °C and with a humidity of 90%. In addition, two sealed conditions (denoted as S1 and S2) were implemented to simulate different levels of oxygen supply in the cathode. The S1 condition was the same as mentioned before (four lateral surfaces were fully sealed), while in the S2 condition only the upper half of the four lateral surfaces was sealed.

## 3. Electrochemical Model for Steel Corrosion in Concrete

When the passivation film on the surface of the steel bar is destroyed, the steel bar is in an activated state, and electrochemical corrosion occurs when water and oxygen are sufficient. Due to the isolated anode and cathode regions on the surface of the corroded steel bar, the transfer of electrons during the corrosion reaction flows through the anode region of the steel bar to the cathode region, thereby causing a corrosion current. According to the Nernst equation [1], the equilibrium potentials of the anode and cathode in steel corrosion reactions are calculated as follows:

(2) $$\varphi_{0,\mathrm{Fe}}=\varphi_{\mathrm{Fe}}^{\Theta }+\frac{2.3RT}{n_{a}F}\mathrm{lg}[{\mathrm{Fe}}^{2+}]$$

(3) $$\varphi_{{0,O}_{2}}=\varphi_{O_{2}}^{\Theta }+\frac{2.3RT}{n_{c}F}\mathrm{lg}\frac{P_{O_{2}}}{{\left[{\mathrm{OH}}^{-}\right]}^{4}}$$

where $\varphi_{0,\mathrm{Fe}}^{\Theta }$ and $\varphi_{{0,O}_{2}}^{\Theta }$ are the standard electrode potential of iron and oxygen at 25 °C, respectively ($\varphi_{0,\mathrm{Fe}}^{\Theta }=-0.44\;V,\;\varphi_{{0,O}_{2}}^{\Theta }=0.401\;V$), *R* is the gas constant (*R* = 8.314 J/K), *T* is the temperature (in K), *n*_a_ and *n*_c_ are the numbers of electrons in anodic and cathodic reactions, respectively (*n*_a_ = 2, *n*_c_ = 4), *F* is the Faraday constant (*F* = 96,500 C/mol), [Fe^2+^] is the Fe^2+^ concentration around steel bars (in mol/L), $P_{o_{2}}$ is the partial pressure of oxygen in concrete pore fluid under an equilibrium state ($P_{o_{2}}=0.21\times 10^{-5}\;\mathrm{Pa}$), and [OH^−^] is the OH^−^ concentration in the concrete pore solution (in mol/L).

Due to polarization, the potentials of the cathode and anode will be close to each other. At a steady state, the anode and cathode reach the same potential, namely the corrosion potential, and the current intensity between the anode and cathode is equal, that is, the corrosion current. The corrosion current can be calculated by

(4) $$\varphi_{0{,O}_{2}}+\eta_{c,e}+\eta_{c,d}-\left(\varphi_{0,\mathrm{Fe}}+\eta_{a,e}\right)=I_{\mathrm{corr}}\cdot R$$

where *η*_c,e_ and *η*_c,d_ are the electrochemical polarization overpotential and concentration polarization overpotential at the cathode, respectively, *η*_a,e_ is the anodic electrochemical polarization overpotential, *I*_corr_ is the corrosion current, and *R* is the concrete resistance between the anode and cathode.

According to the steel corrosion theory [1], the polarization overpotentials for the anode and cathode are given by

(5) $$\eta_{a,e}=b_{a}\mathrm{lg}\frac{i_{a}}{i_{a}^{0}}$$

(6) $$\eta_{c,e}=b_{c}\mathrm{lg}\frac{i_{c}}{i_{c}^{0}}$$

(7) $$\eta_{c,d}=\frac{2.3RT}{n_{c}F}\mathrm{lg}\left(1-\frac{i_{c}}{i_{c,L}}\right)$$

where *b*_a_ is the Tafel slope of the anode (for depassive steel bars in concrete, *b*_a_ = 90.7 mV/decade, and *b*_a_ is infinite for passive steel bars), *b*_a_ is the Tafel slope of the cathode (*b*_c_ = −176.3 mV/decade), *i*_a_ and *i*_c_ are the corrosion current density for the anode and cathode, respectively, $i_{a}^{0}$ and $i_{c}^{0}$ are the exchange current density for the anode and cathode, respectively ($i_{a}^{0}=2.75\times 10^{-8}\;A/{\mathrm{cm}}^{2}$,$\;i_{c}^{0}=6\times 10^{-10}\;A/{\mathrm{cm}}^{2}$), and *i*_c,L_ is the limit current density of the cathode regarding O_2_ diffusion, which is given by

(8) $$i_{c,L}=\frac{n_{c}FD_{c}[O_{2}]}{\delta }$$

where *D*_c_ is O_2_ diffusivity in concrete which is determined by concrete porosity and RH [36], [O_2_] is O_2_ concentration in a concrete pore solution, and *δ* is the thickness of the Nernst diffusion layer, which approximately equals to concrete cover thickness.

Combining Equations (4)–(7) and considering the cathode and anode areas of steel bars yield the equations for the corrosion current density for the anode and cathode:

(9) $$\{\begin{array}{c} \varphi_{0{,O}_{2}}+b_{c}\mathrm{lg}\frac{i_{c}}{i_{c}^{0}}+\frac{2.3RT}{n_{c}F}\mathrm{lg}\left(1-\frac{i_{c}}{i_{c,L}}\right)-\left(\varphi_{0,\mathrm{Fe}}+b_{a}\mathrm{lg}\frac{i_{a}}{i_{a}^{0}}\right)=I_{\mathrm{corr}}\cdot R\\ I_{\mathrm{corr}}=i_{a}\cdot S_{a}=i_{c}\cdot S_{c} \end{array}$$

When the ambient RH is greater than 90%, the diffusion of oxygen becomes the key to control the whole electrochemical corrosion process, and the steel corrosion rate *i*_corr_ approximately equals to the limit current density *i*_c,L_. It is assumed that the oxygen diffuses along the path connecting any positions on the concrete surface to the steel surface. The amount of oxygen diffused to the surface of the steel element per unit time is expressed in the following polar coordinate:

(10) $$\pi =-\int_{\alpha }^{\beta }D_{c}\frac{{\left[O_{2}\right]}_{\mathrm{cl}}-{\left[O_{2}\right]}_{\mathrm{ss}}}{\left(c+r\right)/\cos\theta -r}rd\theta$$

where *α* and *β* are two parameters related to the steel location, [O_2_]_cl_ and [O_2_]_ss_ are the oxygen concentration on the concrete surface and steel surface, respectively, and *c* is the concrete cover thickness.

When the oxygen concentration dominates the electrochemical process, the oxygen reaching the steel surface will be immediately consumed. Thus, the oxygen concentration on the cathode steel surface is assumed to be zero. According to Faraday’s law, the oxygen amount from Equation (10) was used to calculate the following corrosion rate:

(11) $$i_{\mathrm{corr}}=\lambda n_{c}F\int_{\alpha }^{\beta }D_{c}\frac{{\left[O_{2}\right]}_{\mathrm{cl}}-{\left[O_{2}\right]}_{\mathrm{ss}}}{\left[\left(c+r\right)/\cos\theta -r\right]\cdot S}rd\theta$$

where *λ* is a parameter considering the solubility of O_2_ in a pore solution, and *S* is the surface area of steel.

## 4. Results and Discussion

### 4.1. Strength and Porosity

**Figure 2:**
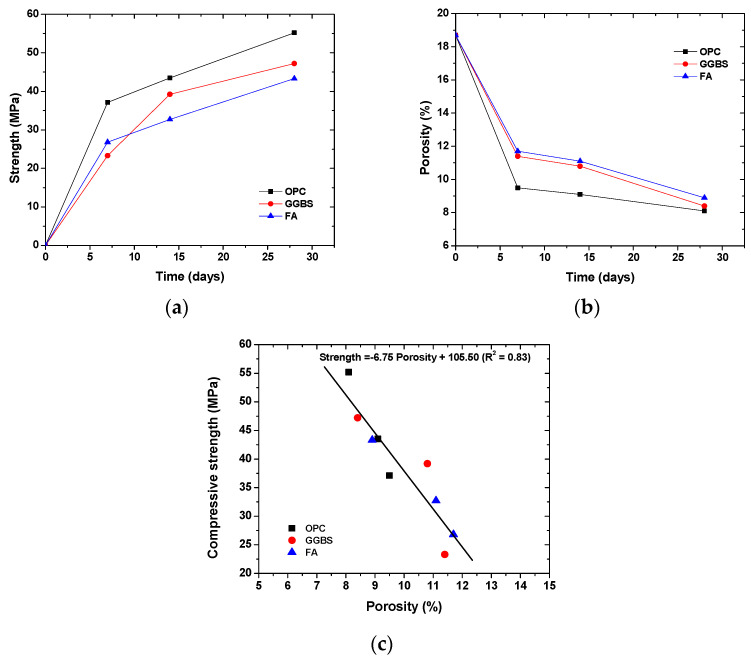
Strength and porosity in concrete: (**a**) time-dependent evolution of compressive strength; (**b**) time-dependent evolution of porosity (the initial porosity, i.e., the porosity at 0 day was calculated based on the mix proportion); (**c**) correlation between compressive strength and porosity.

Figure 2a,b show the development of the strength and porosity in concrete, and Figure 2c shows the correlation between strength and porosity. It can be seen that the concrete gained most strength at 7 days and had the strongest reduction in porosity at 7 days, which was similar for all binder types. The addition of slag and fly ash decreased the strength but increased the porosity within 28 days, which is attributed to the slow pozzolanic reaction [37]. In addition, the compressive strength was negatively correlated to the porosity, irrespective of the binder types. This finding justifies the prediction that the compressive strength of concrete is based on the porosity [38,39].

### 4.2. Carbonation and Chloride Resistance

**Figure 3:**
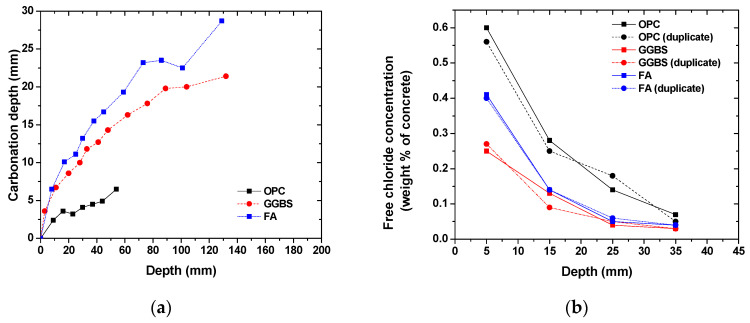
Carbonation and chloride resistance of concrete: (**a**) time-dependent evolution of carbonation depth; (**b**) depth-dependent profiles of the free chloride concentration.

Figure 3 shows the time-dependent evolution of the carbonation depth and free chloride concentration profiles in concrete with various binder types. As expected, the carbonation depth increased over time but at a much faster rate for concrete with slag and fly ash incorporation [40]. The carbonation depth of slag and fly ash concrete was up to 6 times higher than that of OPC concrete. The reduction in the carbonation resistance in slag and fly ash concrete is attributed to the low-calcium content in the cementitious binder due to the dilution effects and pozzolanic reaction. The reactive aluminosilicate component in the glassy structure of slag and fly ash can react with the portlandite, thus decreasing the carbonation resistance [40]. In addition to the formed calcium-silicate-hydrate due to pozzolanic reactions, the hydration of slag and fly ash can result in an AFm-type phase and alumina-modified calcium-silicate-hydrate formation. The formed AFm phase, a type of Ca–Al-layered double hydroxide, can undergo an ionic exchange reaction between the hydroxide and chloride ions, thus increasing the chloride binding capacity [41,42,43]. Therefore, it is reasonable to observe the improved chloride resistance in concrete incorporated with slag and fly ash. Although the porosity was slightly reduced in concrete due to slag and fly ash incorporation, the more refined pore structure in slag and fly ash concrete could contribute to a reduction in chloride penetration [37,43,44]. As listed in Table 2, the pH value measured in concrete with slag and fly ash was slightly smaller than that of OPC concrete, which was attributed to the enhanced alkali binding in alumina-modified calcium-silicate-hydrate [45]. However, the pH at the concrete surface after carbonation was similar for all concrete groups.

### 4.3. Corrosion Resistance

**Figure 4:**
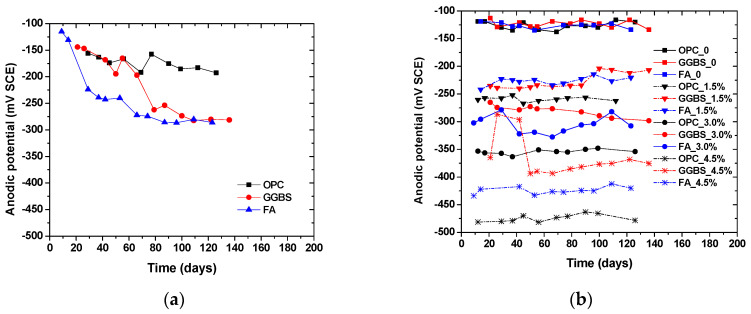

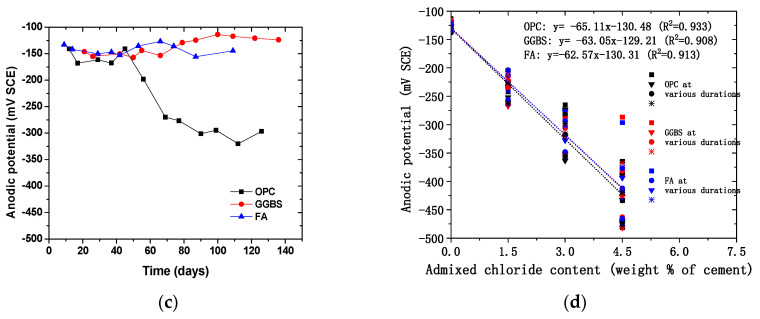
Time-dependent evolution of corrosion potential in the top steel (anode) embedded in concrete exposed to (**a**) accelerated carbonation; (**b**) chloride contamination; (**c**) chloride penetration; (**d**) the correlation between admixed chloride content and the corrosion potentials.

**Figure 5:**
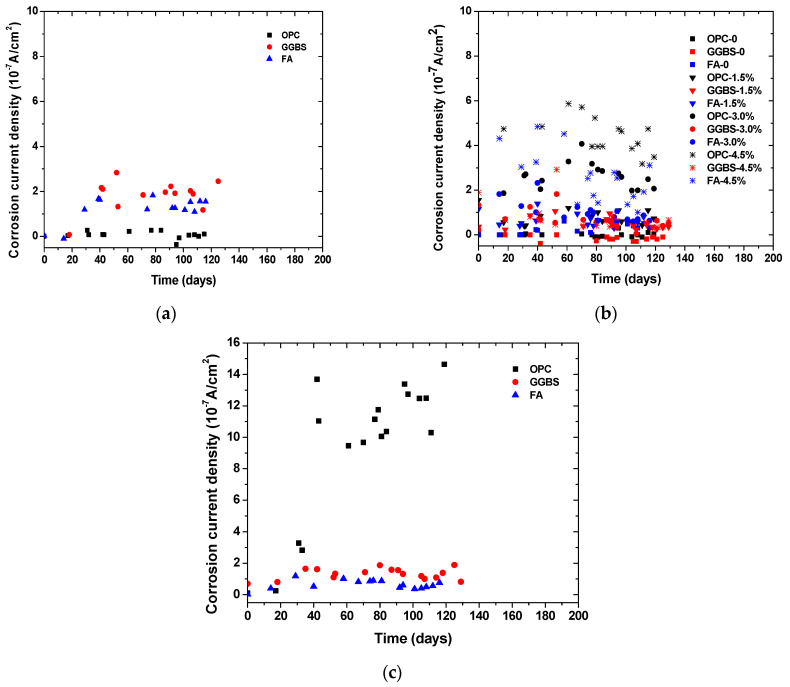
Time-dependent evolution of corrosion current density in concrete exposed to (**a**) accelerated carbonation; (**b**) chloride contamination; (**c**) chloride penetration.

Figure 4 and Figure 5 show the time-dependent evolution of the anodic potentials and macrocell corrosion current density of steel bars embedded in concrete exposed to the three simulated environments. In an accelerated carbonation condition, the corrosion potentials became more negative over time, indicating the depassivation and gradual increase in the corrosion rate of steel. According to the criterion for steel depassivation [46], the steel bar is not corroded as the potential of the steel bar is greater than −120 mV, and the probability of steel corrosion is larger than 90% as the potential of the steel bar is less than −270 mV. For the OPC group, the carbonation depth did not exceed the concrete cover thickness when the carbonation time reached 120 days. The anodic potential remained between −100 and −200 mV, and the average current density was 1.5 × 10^−8^ A/cm^2^. The comparison between the actual steel corrosion observation and the corrosion current density in reference [47] indicated that the steel bar was obviously in a passive state when the current density was about 4 × 10^−8^ A/cm^2^. Therefore, it can be concluded that the upper steel of the OPC group is in a passive state. For the FA group, the anodic potential shifted negatively from −130.7 mV on the 14th day to −272.3 mV on the 66th day, and the corrosion current density increased from −1.0 × 10^−8^ A/cm^2^ on the 14th day to 1.67 × 10^−7^ A/cm^2^ on the 39th day, indicating the initiation of steel corrosion. For the GGBS group, the corrosion current density increased from 0.7 × 10^−8^ A/cm^2^ on the 18th day to 2.16 × 10^−7^ A/cm^2^ on the 41st day, indicating steel corrosion, although the steel potential did not drop below −270 mV. This is because anodic potential is an index for the possibility of steel depassivation, but the current density can directly reflect the evolution of the corrosion rate. With the same carbonation duration, the fly ash concrete showed the lowest potential, followed by slag and OPC concrete, which is in strong agreement with the observed carbonation depth evolution. The low amount of calcium in fly ash in comparison to slag exacerbated the carbonation resistance even at a lower replacement level. The corrosion current density of fly ash and slag concrete tended to increase over time, while OPC concrete remained at a low value. This is in agreement with Andrade and Buják’s results [48] that the corrosion rate was lower for OPC than that for supplementary cementitious materials. However, some results showed the corrosion rate for OPC was higher than that with clinker replacement at a low RH [5].

In the case of internally admixing chloride, it can be seen that the corrosion potential was significantly dependent on the amount of admixed chloride salts, irrespective of binder types. The anodic potential for the group without admixing chloride ions remained at about −120 mV, indicating that the steel was not corroded. The correlation between the amount of admixed chloride salts and measured potentials showed a linear trend, as shown in Figure 4d. Although insignificant, the corrosion potential tended to slightly increase (more positive) over time due to chloride binding (conversion of admixed free chloride into physical or chemical bound chloride). It is also reasonable to observe that the corrosion current density of steel was higher at a larger chloride admixture content. At the same chloride content, the corrosion potential drop and corrosion current density increase tended to be lower for concrete with slag and fly ash than those of OPC concrete, which might be attributed to the enhanced chloride binding. According to the previous studies, the incorporation of slag and fly ash can considerably improve the chloride binding capacity of cement due to the enhanced formation of AFm and hydrotalcite-type phases [41,42,43].

Chloride penetration into the concrete around the upper reinforcement was accelerated by the drying and wetting cycles. When the chloride ion content on the steel surface reaches a critical value, the passive film on the surface of the steel bar is destroyed, and the potential of the steel bar decreases rapidly, at which point corrosion initiation occurs. Sandberg’s result [49] showed that the periodic wetting and drying conditions could also reduce the critical chloride value for the corrosion initiation. The corrosion potential of OPC concrete dropped considerably over time while those of slag and fly ash concrete remained relatively constant. At around 40 days of exposure, the corrosion potential of OPC concrete suddenly dropped and the corrosion current density increased dramatically as well. This finding is consistent with the free chloride profiles result, which indicates a significant improvement of the chloride penetration resistance of concrete due to slag and fly ash incorporation. It was found that the sudden change occurred earlier in the current density than that in the anodic potential. This is because the area ratio of cathode to anode is large at the start of corrosion causing the current density to be more sensitive to corrosion initiation. Another reason is that the ingress of chloride ions leads to a decrease in concrete resistivity, which reduces the polarization of resistance.

It can be seen that, regardless of the simulated environments, the initial corrosion potential of the anode steel was around −120 mV (SCE). Depending on the exposure condition and binder types, the corrosion potential dropped and corrosion current increased since the depassivation of steel occurred. However, the extent of the potential drop and the corrosion current increase was dependent on the simulated environments. For example, although the potential dropped significantly for the concrete with more than 1.5% salt contamination, its corrosion current density was about 50% lower than the concrete under external chloride penetration at a similar chloride content. It suggests that drying–wetting cycles can exacerbate corrosion development in concrete, probably due to the presence of microcell corrosion in the top layer of steel, in addition to the induced macrocell corrosion. In contrast, the internal chloride admixing at the top layer of steel ensured full-scale depassivation and thus prevented the formation of additional cathode zones in the top steel. In addition, by comparing the concrete exposed to carbonation and chloride penetration, it can be seen that at the same level of corrosion potentials, the current rate was significantly higher in the case of chloride-induced corrosion. The finding suggests that the correlation between corrosion potential and corrosion current density is dependent on exposure conditions.

### 4.4. Effect of Geometrical Parameters and Boundary Conditions

**Figure 6:**
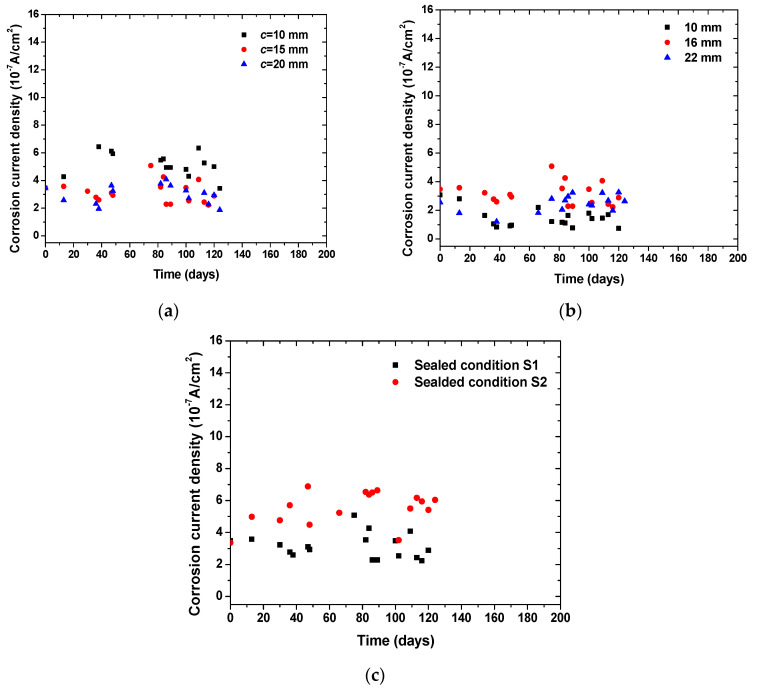
Time-dependent evolution of corrosion current density in concrete specimens with various geometrical parameters and boundary conditions: (**a**) effect of cover thickness; (**b**) effect of steel diameter; (**c**) effect of sealed condition.

Figure 6 shows the time-dependent evolution of macrocell corrosion current density in concrete specimens with various concrete cover thickness, steel diameters, and sealed conditions. It can be seen that an increase in concrete cover thickness tended to decrease the corrosion rate of steel at the same level of chloride content and anodic potentials. It should be noted that the thickness of concrete cover was increased for the bottom steels (cathode) but not for the top steel (anode). As designed in the specimens, the two bottom steels took oxygen and underwent cathode reaction, while the released electron was transported to the top steel for anode reaction. The reduction in the corrosion rate suggests that the supply of oxygen in the cathode played an important role in the macrocell corrosion reaction. The increase in steel diameter tended to increase the corrosion rate, which is attributed to the enlarged contact surface of steel with concrete. In addition, it can be seen that the specimens sealed with the S2 condition showed a higher corrosion rate than that of the specimens sealed with the S1 condition. As the S2 condition had a higher exposure surface area of concrete to the atmosphere than the S1 condition, this allowed for a high amount of oxygen supply to the cathode. This testing suggests that oxygen supply played a critical role in determining the macrocell corrosion kinetics of steel embedded in concrete.

### 4.5. Comparison between Measured and Modeled Results

For the results in Section 4.3, the corrosion current density was modeled by substituting appropriate parameters into Equation (9). For carbonated concrete, OH^−^ concentration in the pore solution was taken as 1 × 10^−5.5^ mol/L; otherwise, it was given as 1 × 10^−1.5^ mol/L. The equilibrium potentials of the cathode were calculated according to Equation (3) while the equilibrium potentials of the anode were taken as measured values. By using an empirical model for concrete resistivity [50], the resistivity between the anode and cathode for OPC, GGBS, and FA concrete groups are given as 2605, 4656, and 3361 Ω, respectively. In the complete depassivation stage, the area ratio of anode to cathode is taken as 1:2. The modeled corrosion current density was compared with the averaged values for different environmental conditions, as shown in Table 3. The carbonation depth of the OPC group did not reach the steel surface, and the upper reinforcement was passive without any corrosion current density. The results for internally admixed chloride groups showed that the model error was greatest when GGBS was used as an admixture in concrete. This is because 50% GGBS replacement increases the chloride capacity of concrete greatly, reducing the free chloride ion content in the pore solution. The reduction in free chloride concentration leads to the increase in concrete resistivity, and thus the measured values were smaller than the modeled ones for the GGBS group. For the chloride penetration group, the measured density was larger than the modeled value. This can be explained by the reduction in concrete resistivity due to more absorption of water and chloride under periodic wetting and drying conditions. This indicated that water and chloride transport models should be combined with the corrosion rate model when corrosion caused by chloride penetration is considered.

For the results in Section 4.5, a high RH was selected as an environmental condition, so the electrochemical process became oxygen-concentration controlled and Equation (11) was used to calculate the corrosion current density. The modeled corrosion current density was compared with the average of the measured values, as shown in Table 4. It was found that the error was relatively large for the steel bar diameter of 10 mm. The reason is that the steel surface area is relatively small, and the iron rust generated by anodic reaction is more likely to block the pores of concrete with the progress of corrosion, which hinders the diffusion of hydrated iron ions and reduces the corrosion rate. Therefore, the oxygen diffusion model established in this paper is not suitable for steel bars with a diameter smaller than 10 mm.

## 5. Conclusions

In this paper, the strength, carbonation and chloride penetration resistance, and macrocell corrosion behaviors of steel embedded in concrete exposed to carbonation, chloride contamination, and chloride penetration were studied. In particular, a comparison was made between the corrosion resistance of concrete prepared with various binder types, exposure environments, specimen geometrical parameters, and boundary conditions. The following conclusions can be drawn based on this study:

1. The addition of slag and fly ash significantly enhances the chloride-induced corrosion resistance but undermines the carbonation-induced corrosion resistance. Internal chloride admixing has less detrimental effects on concrete incorporated with slag and fly ash than pure Portland cement concrete, due to the enhanced binding of admixed chloride ions.
2. The macrocell current density due to concrete carbonation is obtained. For the OPC group, the embedded steel bar remains passive, and the average current density is about 1.5 × 10^−8^ A/cm^2^. The steel depassivation for the FA and GGBS groups occurs, and the average corrosion current density in the corrosion propagation stage is 1.40 × 10^−7^ A/cm^2^ and 2.05 × 10^−7^ A/cm^2^, respectively.
3. Although the corrosion potentials can be qualitatively correlated to the macrocell corrosion current density, this correlation is dependent on the exposure conditions. The drying–wetting cycle is more detrimental than the chloride contamination in terms of corrosion acceleration at a similar chloride content in concrete. At the same level of potential drops, the chloride-induced corrosion shows a higher rate than that of carbonation-induced corrosion.
4. The corrosion rate model of steel bars based on two-dimensional oxygen diffusion is verified by experiments. Compared with one-dimensional diffusion, two-dimensional diffusion can more accurately reflect the cathodic reaction process on corroded steel bars in practical engineering and provide a theoretical basis for considering the effect of steel bar position.

## Figures and Tables

**Table 1:** Mix proportion of concrete.

Parameters	Concrete Group		
	OPC	GGBS	FA
Water-to-binder ratio	0.53	0.53	0.53
Cement (kg/m^3^)	370	185	258
Slag (kg/m^3^)	0	185	0
Fly ash (kg/m^3^)	0	0	110
Water (kg/m^3^)	187	187	186
Sand (kg/m^3^)	749	750	747
Coarse aggregate (kg/m^3^)	1110	1112	1107

**Table 2:** pH value of concrete before and after carbonation.

Condition	Concrete Group		
	OPC	GGBS	FA
28-day moist curing	13.34	12.75	12.59
112-day carbonation	9.15	9.07	9.14

**Table 3:** Comparison between measured and modeled corrosion current density (×10^−7^ A/cm^2^) for different environmental conditions.

Conditions	OPC		GGBS		FA	
	Measured	Modeled	Measured	Modeled	Measured	Modeled
Carbonated	-	-	2.05	2.16	1.40	1.46
Internally admixed with 1.5% NaCl	0.78	1.44	0.52	1.20	0.66	1.02
Internally admixed with 3% NaCl	2.62	2.31	0.68	1.62	1.03	1.45
Internally admixed with 4.5% NaCl	4.46	3.71	0.71	2.61	2.60	3.09
Chloride penetration	11.72	2.22	-	-	-	-

**Table 4:** Comparison between measured and modeled corrosion current density (×10^−7^ A/cm^2^) for different geometrical parameters and boundary conditions.

Conditions	Measured	Modeled
Cover thickness		
10 mm	5.21	4.67
15 mm	3.16	3.03
20 mm	3.00	2.17
Steel bar diameter		
10 mm	1.48	3.14
16 mm	3.16	3.03
22 mm	2.69	2.92
Sealing of lateral surfaces		
Fully sealed	3.16	3.03
Upper half sealed	5.53	6.05

## Data Availability

The data presented in this study are available on request from the corresponding author. The data are not publicly available due to privacy restrictions.
